# Adsorption of Metal Ions from Single and Binary Aqueous Systems on Bio-Nanocomposite, Alginate-Clay

**DOI:** 10.3390/nano14040362

**Published:** 2024-02-15

**Authors:** Rachid Aziam, Daniela Simina Stefan, Safa Nouaa, Mohamed Chiban, Magdalena Boșomoiu

**Affiliations:** 1 Laboratory of Applied Chemistry and Environment, Department of Chemistry, Faculty of Science, Ibnou Zohr University, Agadir BP 8106, Morocco; rachid.aziam@edu.uiz.ac.ma (R.A.); safa.nouaa@edu.uiz.ac.ma (S.N.); m.chiban@uiz.ac.ma (M.C.); 2 Department of Analytical Chemistry and Environmental Engineering, Faculty of Chemical Engineering, and Biotechnologies, National University of Science and Technology Politehnica of Bucharest, 1-7 Polizu Street, 011061 Bucharest, Romania; daniela.stefan@upb.ro

**Keywords:** bio-nanocomposite, alginate, heavy metals, natural moroccan clay

## Abstract

The aim of this work is to characterize and evaluate the retention of Cu^2+^ and Ni^2+^ from single and binary systems by alginate-Moroccan clay bio-composite with the utilization of calcium chloride as a cross-linking agent, using the ionotropic gelation method. The bio-nanocomposite was characterized by using a variety of techniques (SEM, EDX, XRD, and pH_PZC_). The efficiency of the adsorbent was investigated under different experimental conditions by varying parameters such as pH, initial concentration, and contact time. To demonstrate the adsorption kinetics, various kinetic models were tried and assessed, including pseudo-first-order, pseudo-second-order, intraparticle diffusion, and Elovich models. The research results show that the adsorption process of Cu^2+^ and Ni^2+^ metal ions follows a pseudo-second-order kinetic model, and the corresponding rate constants were identified. To evaluate the parameters related to the adsorption process in both single and binary systems, different mathematical models of isotherms, such as Langmuir, Freundlich, Temkin, and Dubinin-Radushkevich, were investigated. The correlation coefficients obtained showed that the most suitable isotherm for describing this adsorption process is the Langmuir model. The process is considered to be physical and endothermic, as suggested by the positive values of Δ*H°* and Δ*S°*, indicating increased randomness at the solid/liquid interface during Cu^2+^ and Ni^2+^ adsorption. Furthermore, the spontaneity of the process is confirmed by the negative values of ∆*G*°. The bio-nanocomposite beads demonstrated a maximum adsorption capacity of 370.37 mg/g for Ni^2+^ and 454.54 mg/g for Cu^2+^ in the single system. In the binary system, the maximum adsorption capacities were observed to be 357.14 mg/g for Ni^2+^ and 370.37 mg/g for Cu^2+^. There is significant evidence for the use of alginate-Moroccan clay bio-nanocomposite as a cost-effective alternative adsorbent for the efficient removal of metal ions in single and binary systems.

## 1. Introduction

One phenomenon that has attracted worldwide concern is heavy metal contamination, which is now a significant environmental issue and a major stress. Due to their toxicity, bioaccumulation, and non-biodegradability, these pollutants are of particular interest to researchers who study human health and aquatic environments [1,2]. These pollution problems are the result of rapid growth in industrial activity which has resulted in an environmental disorder [1]. 

As stated by Tchounwou et al. (2012) [3], heavy metals are classified as metallic elements with a density that is very high when compared to water. They can be identified by having a high atomic weight, and a density that is around five times more than that of water. They are present in a variety of industrial effluents produced by various human activities, including tanneries, mineral extraction, and plating facilities, and they are also continuously released into the environment by volcanoes, as a result of rocks naturally weathering [2,4,5]. 

Metal toxicity leads to the creation of free radicals, which cause DNA damage [6]. The formation of free radicals has been studied in particular for iron, copper, nickel, chromium, and cadmium. The last metals are known for their carcinogenic properties. We have chosen to investigate two metals in this study, namely, nickel and copper. These metals have been selected because of their extreme toxicity, multitude of information available regarding their biogeochemical cycles, and the fact that they are on lists of priority metals for monitoring contamination in rivers and marine waters [7].

Copper is a type of inorganic pollutant that has been extensively studied by researchers in the field of bio-absorption [8,9]. According to Kadirvelu et al. (2000) [10], this metal can exist in the form of the free cation Cu^2+^ in acidic environments, and as traces of soluble Cu(OH)_2_ and [Cu(OH)]^+^ in neutral or basic environments. In order to address the adverse impacts of heavy metals, environmental agencies establish acceptable thresholds for their concentrations in various types of water, including drinking water. As an illustration, the World Health Organization [11] stipulates a maximum allowable concentration of 1 mg/L for copper.

Nickel is used in stainless steel currency, metallic alloys, super alloys, nonferrous metals, mineral processing, paint formulation, electroplating, battery manufacturing, and copper sulfate manufacturing [12]. In addition to being a heavy metal ion that is frequently used, nickel is also toxic. Its toxicity spreads throughout the chemical, electroplating, mining, refining, paint, and ink formulation sectors [13]. It has detrimental consequences on health, including cancer, dermatitis, nausea, persistent asthma, and coughing. The maximum amount of nickel allowed in the drinking water is 0.015 mg/L, according to the US EPA [14].

Wastewaters that contain copper and nickel should be appropriately treated before being released, due to their toxicity.

The conventional techniques for removing Ni^2+^ and Cu^2+^ ions from aqueous solutions include ion exchange, solvent extraction, chemical precipitation, oxidation/reduction, filtration, reverse osmosis, membrane technology, and adsorption methods [15,16,17,18,19,20]. Considering it is less expensive and easier to understand, design, and operate, the adsorption process appears to be a more suitable approach for controlling water pollution [21].

Other methods used for removing hazardous metals from dissolved solutions include electrodialysis and precipitation [22,23,24].

Recently, there has been a focus on using alternative, low-cost materials as potential adsorbents for the removal of hazardous metals [25]. Many research investigations have examined the retention of metal ions on various adsorbents, including sodium aluminosilicate, activated carbon, zeolites, clays, and metal oxides [25,26,27,28,29,30,31,32,33,34].

Filice et al. (2021) [35] and Filice et al. (2022) [36] have demonstrated the efficiency of a Halloysite-type clay for water purification in general and the removal of organic and inorganic pollutants in particular. The removal efficiency of natural clay is higher than most conventional adsorbents, and it has been tested as novel nanomaterial that can be used in water purification.

Clays are highly efficient and selective materials that remove metal ions from water; nevertheless, because of their high surface area, they agglomerate rapidly and can be difficult to extract from aqueous solutions. Alginate is a naturally occurring, non-toxic, inexpensive, and ecologically friendly polysaccharide with a high degree of biodegradability that is used as a biopolymeric support for clay [37,38].

According to the findings by Zhao et al. (2023), [39] coordination polymers prove to be highly suitable for detecting minute concentrations of environmental toxins. This is attributed to their favourable characteristics, including ease of production, swift response, and heightened sensitivity.

The purpose of this work is to assess the feasibility of applying alginate-Moroccan clay beads to eliminate toxic heavy metals in aqueous solution. The effects of experimental conditions such as contact time, metal ion concentration, and pH were studied. Experimental results have been analyzed to understand the adsorption mechanism. To demonstrate the adsorption kinetics, various kinds of kinetic models were assessed and tested, including the Elovich model, intraparticle diffusion, pseudo-first-order, and pseudo-second-order models. The study findings revealed that the pseudo-second-order kinetic model gave a good description of the removal process of metal ions onto the prepared support, with associated rate constants that were effectively identified. The results achieved revealed that the bio-nanocomposite beads have a high adsorption capacity for the removal of Cu^2+^ and Ni^2+^ in single and binary systems, which is scientifically relevant. The Langmuir isotherm proved to be the most appropriate for describing the interaction between the adsorbent and the metal ions. In accordance with the experimental findings, the physical and endothermic adsorption process is due to the Δ*H°* value. The spontaneous nature of the adsorption is demonstrated by the negative values of *∆G°*. The random nature of the solid/liquid interface that occurs during adsorption is shown by positive Δ*S*° values.

## 2. Materials and Methods

### 2.1. Materials and Preparation of Adsorbent

The clay employed in the study was collected from the Tafraout area, situated in the southern Souss region of Morocco. Sodium alginate (MW—70,000–80,000) used in this study is derived from brown algae, and is a polysaccharide consisting of repetitive units of mannuronic acid and guluronic acid sugars. These sugar components contribute to the distinctive properties of alginate. It was obtained from Himedia, India. The chemicals utilized included calcium chloride (CaCl_2_) and mono-component aqueous solutions of metals, prepared from the following corresponding salts: CuSO_4_ and NiCl_2_ (all p.a. Fluka). All reagents were of analytical grade and used as received. The pH of the solution was adjusted using 0.1 M HCl and NaOH solutions, prepared using deionized water. The prepared clay/alginate ratio was 2:1 using the extrusion synthesis process; a bio-nanocomposite made from Moroccan clay encapsulated in alginate was developed. The alginate was continuously dispersed with double-distilled water in a 100 mL Erlenmeyer flask for seven hours at 40 °C. The suspension was then mixed with natural Moroccan clay under gentle magnetic stirring at room temperature. The solution was stirred at 500 rpm to ensure complete homogenization of the alginate and clay and was introduced into a syringe to produce beads.

The syringe was placed vertically above a gelling solution of 0.1 M calcium chloride (CaCl_2_). Gradually, the treatment was drip fed into the gel bath. The saline solution gels quickly, and the creation of chains around the Ca^2+^ cations result in the formation of beads [38].

### 2.2. Batch Adsorption Studies

In the batch adsorption experiments, a volume of 50 mL with the initial concentration *C*_0_, was mixed with 0.05 g of prepared alginate-clay beads. The mixture was stirred well with the use of a magnetic stirrer and was placed in a water bath thermostat to keep a constant temperature.

After the adsorption process had been completed within a contact time (t), the obtained solutions were centrifuged at 5000 rpm for 10 minutes, and subsequently analyzed. The residual concentration of metal ions (Cu^2+^ and Ni^2+^) in the supernatant was determined by flame atomic absorption spectroscopy (Analytik Jena ContrAA 300, Bucharest, Romania).

The retention of Cu^2+^ or Ni^2+^ ion concentrations (*C_r_*–removal concentration) from the aqueous solution was determined as the difference between the initial concentration (*C*_0_, mg/L) and the residual concentration at different contact times (*C_t_,* mg/L). The adsorbed quantity (*q_t_*, mg/g) at time “*t*” was calculated as described below:

(1) $$q_{t}=\left(C_{0}-C_{t}\right)\times \frac{V}{m}$$

where *V* (L) is the volume of the solution and *m* (g) is the adsorbent dose.

The removed rate of Cu^2+^ and Ni^2+^ ions was determined by calculating the following:

(2) $$\%adsorption=\frac{100(C_{0}-C_{t})}{C_{0}}$$

### 2.3. Characterization of Bio-Nanocomposite Beads

#### 2.3.1. Morphology Analysis and Specific Surface Area

To understand the structure sight of the alginate-Moroccan clay bio-nanocomposite beads, scanning electron microscopy (SEM) was generally employed to visualize the samples’ morphology. The microparticles’ structure and morphology were characterized by scanning electron microscope, SEM, using Quanta Inspect F50, FEI Company, Eindhoven, Netherlands, which was equipped with a field emission electron gun (FEG)—with a resolution of 1.2 nm, and an energy dispersive X-ray spectrometer (EDS) with a resolution of MnK of 133 eV. The specific surface area was measured using a nitrogen adsorption-desorption technique; the samples were outgassed at 40 °C, 17 h before recording N_2_ adsorption-desorption isotherms (Micromeritics, TriStar II Plus). The specific surface area was determined in the relative pressure range, P/P_0_ of 0.08–0.25 using Brunauer-Emmett-Teller (BET) theory. The muscovite sample has a BET specific surface area of 4.9 m^2^/g. The nanocomposite has a very low specific surface area, probably with pores of a diameter higher than 500 nm.

#### 2.3.2. Elemental Analysis

The elemental analysis of alginate-Moroccan clay beads was analyzed by SEM coupled with energy dispersive X-ray analysis (SEM/EDX, Quanta Inspect F50, FEI Company, Eindhoven, The Netherlands).

#### 2.3.3. pH of Point Zero Charge (pH_PZC_) and Zeta Potential for the Bio-Nanocomposite Beads

To determine the pH_PZC_, 0.5 g of the adsorbent was introduced into different 250 mL Erlenmeyer flasks with 50 mL of 0.01 M NaCl solution. The pH values of these solutions varied between 2 and 12 using 0.1 M of HCl and NaOH solutions. These flasks were left for 48 h, and the final pH of the solutions was measured. The point of intersection of the final and initial pH curves was determined as pH_PZC_. 

Zeta potential was quantified with a Malvern Zeta-sizer Nano ZS instrument device with a disposable measuring cell. The zeta potential was determined from the electrophoretic particle mobility using the Smoluchowski model. Data are reported as the average of three consecutive measurements over 20 series.

#### 2.3.4. XRD Analysis

The X-ray diffraction analysis of the prepared adsorbent was analyzed using a Bruker CCD-Apex instrument equipped with an X-ray generator (Ni−filtered Cu-Kα radiation) operating at 40 kV and 40 mA. The X-ray diffraction (XRD) technique was used in the scanning range of 5° ≤ 2θ ≤ 80° to confirm the crystal structure of the adsorbent. 

## 3. Results and Discussion

### 3.1. Characterization of Bio-Nanocomposite Beads

**Figure 1:**
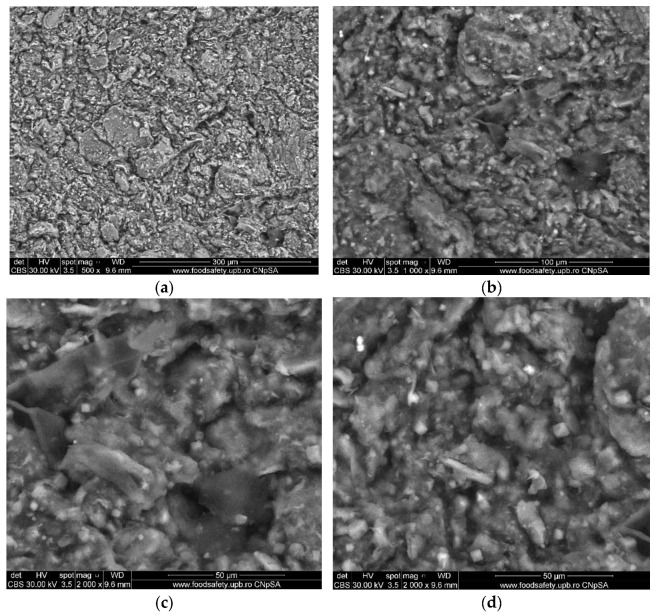
SEM images of alginate-Moroccan clay bio-nanocomposite microparticles: (**a**)–500× magnification level; (**b**)–1000× magnification level; (**c**)–2000× magnification level; (**d**)–2000× magnification level.

The natural clay used in this study was analyzed and characterized by Aziam et al. (2023) [37]. They showed that natural clay can be identified by two aspects, according to SEM images as follows: the distributed powder and the creation of a few agglomerates with varying forms. These agglomerates are created at higher magnifications by the assembling of microscopic particles with varying forms and a heterogeneous morphology. The agglomerate shapes depicted in Figure 1 are distinguished by the substantial number of pores distributed across the entire surface area of the natural clay employed [37].

Figure 1 shows SEM micrographs (a, b, c, and d) of the bio-nanocomposite generated at different levels of magnification. At high magnification, the micrographs illustrate agglomerates of a very fine powder containing particles of different shapes and sizes. SEM micrographs of the bio-nanocomposite beads indicate that the clay sheets are well arranged and dispersed in the matrix. 

**Figure 2:**
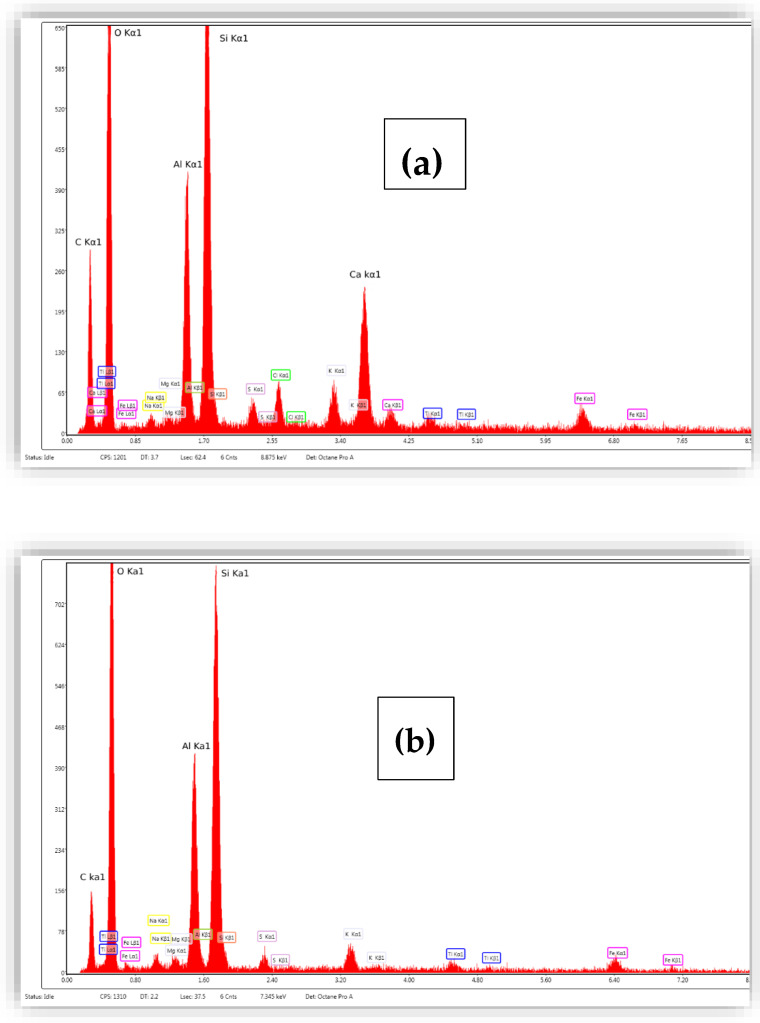
EDX analysis of alginate-Moroccan clay bio-nanocomposite beads (**a**) and natural Moroccan clay (**b**).

EDX is used to analyze the elemental composition of solid samples. The EDX analysis of bio-nanocomposite beads is shown in Figure 2 and Table 1.

EDX analysis of alginate-Moroccan clay bio-nanocomposite beads shows that the average atom fractions of O, C, Si, and Al are approximately 54.04%, 33.83%, 5.45%, and 3.55%, respectively (in atomic percentage %). The appearance of the calcium atom Ca (equal to 1% in atomic %) is due to the addition of alginate, which can contain Ca in its atomic chain from the marine environment. The EDX results for the natural Moroccan clay (Figure 2) indicated the presence of silicon, primarily attributed to the clay mineral and quartz. This observation is corroborated by the XRD results. Moreover, the increased percentage of oxygen and the presence of calcium (Ca) in the EDX peaks of the alginate-Moroccan clay beads suggest the effective integration of sodium alginate into the natural Moroccan clay.

Many studies have demonstrated that the specific surface area of materials used as carriers for metal ion adsorption depends on their particle size [40,41]. The specific surface area was 4.9 m^2^/g for pure clay, while Gupta and Bhattacharyya (2006) [41] found a value of 3.8 m^2^/g. The decrease in specific surface area of the alginate-clay bio-nanocomposite used is around 1 m^2^/g, which can be explained by alginate cross-linking leading to pore filling.

**Figure 3:**
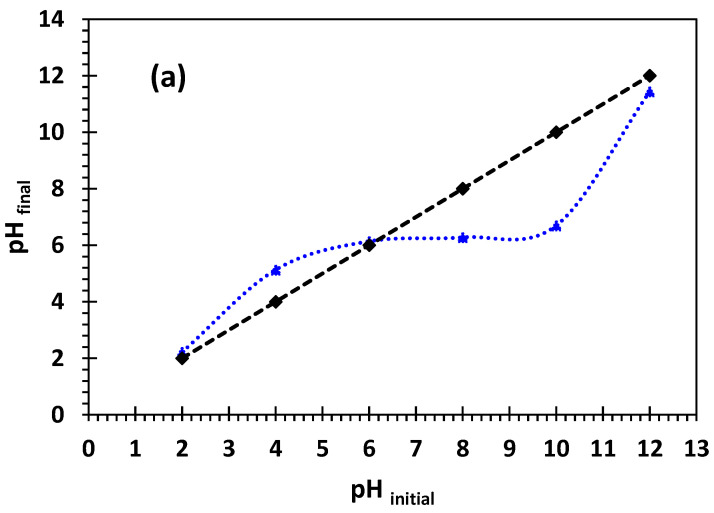

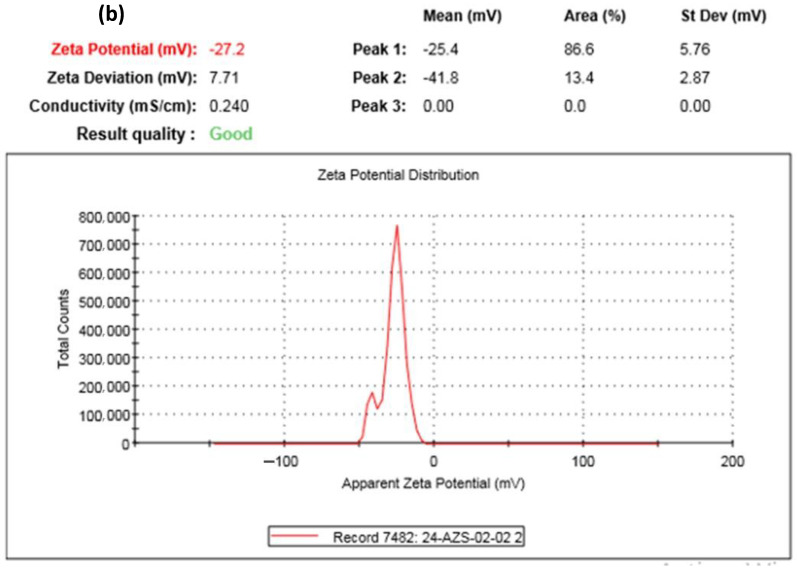
(**a**) pH_PZC_ of the adsorbent, (**b**) zeta potential.

The pH of point zero charge (pH_PZC_) of the adsorbent used was determined to be 6.2 (Figure 3a). This value shows that at a pH less than pH_PZC_, the bio-nanocomposite surface is positively charged, while at a pH higher than pH_PZC_, the surface is negatively charged. Thus, at pH < 6.2, the surface is characterized by a positive charge, confirming that adsorption of positively charged Cu^2+^ and Ni^2+^ metal ions would be limited. At pH > 6.2, with the surface having a high negative charge density, adsorption of Cu^2+^ and Ni^2+^ metal ions becomes significant [21,42,43]. The zeta-potential value of the bioadsorbent particles was −27.2 mV (Figure 3b) attributed to oxygen functions that are negatively charged (clay layers are characterized by the presence of O− in a basic medium).

**Figure 4:**
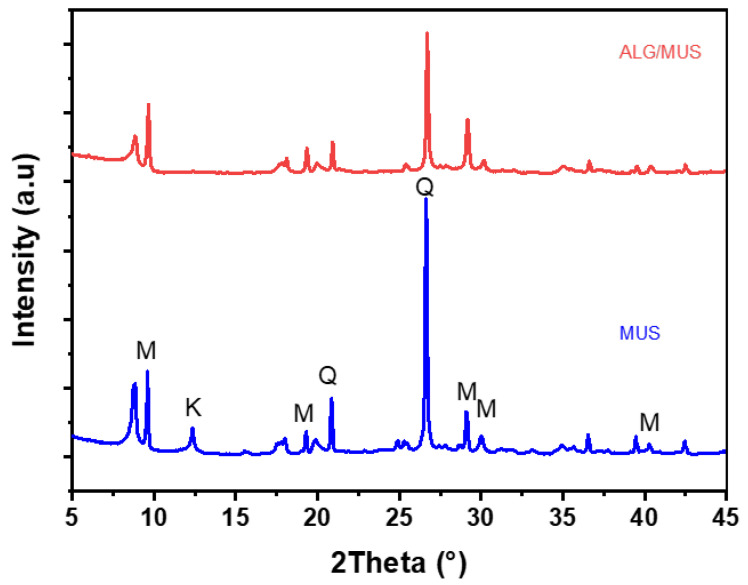
X-ray diffraction: natural Moroccan clay (MUS) and alginate-clay beads (ALG/MUS): (M: muscovite and Q: quartz).

Figure 4 shows the X-ray diffraction spectrum of the adsorbent. Powdered samples were scanned from 5° to 80° (2θ) at a step of 2°/min. X-ray diffraction patterns for the prepared bio-adsorbent are shown in Figure 4. The diffraction signals at 2θ values of 9.61°, 18.05°, 19.85°, 29.13°, 35.03°, and 42.45° correspond to the lattice planes of the clay mineral (muscovite). In addition, there is a diffraction peak at 2θ = 20.95°, 26.68°, and 42.60°, denoting the presence of quartz [44,45]. Diffractograms of the adsorbent reveal the successful dispersion of the clay layers in the amorphous alginate (ALG) matrix. This dispersion is apparent from the displacements observed and the decrease in peak intensity, characteristics typically linked to the interbasal distances between the clay layers [38,46].

### 3.2. Single Component Systems Adsorption of Heavy Metal Ions by the Bio-Nanocomposite Beads

#### 3.2.1. Determination of m/V Ratio

**Figure 5:**
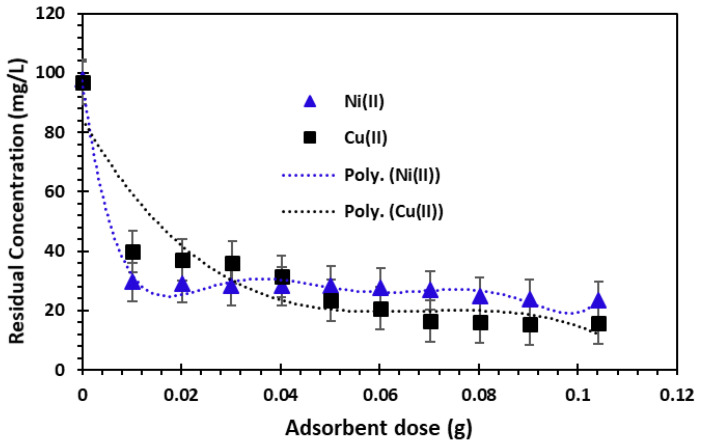
Effect of adsorbent dose on the removal of Cu^2+^ and Ni^2+^ cations (*C*_0_ = 100 mg/L, *T* = 23 ± 2 °C and *T_c_* = 12 h; error bars show means ± standard error from the mean of duplicate experiments).

The ratio between the adsorbent mass and solution volume is an important parameter in the adsorption mechanism. Various masses (m) of bio-nanocomposite beads were stirred with a volume equivalent to 50 mL of metal ion solutions with an initial concentration equal to 100 mg/L for a contact time equivalent to 12 h. The variation in the Cu^2+^ and Ni^2+^ cations’ adsorbed amounts with adsorbent mass is depicted in Figure 5.

The capacity to eliminate the metal cations in question increases with increasing adsorbent dosage, due to a higher number of sites available for the adsorption [45]. In fact, the residual concentration decreases as the mass of the material increases, as shown in Figure 5; at a mass of 0.05 g or more, equilibrium is reached. The amount adsorbed also decreased with an increasing adsorbent mass, as a result of the retention capacity of the adsorbent’s active surface for copper and nickel ions. As the adsorbent dosage was added, the amount adsorbed continued to decrease until it stabilized. Consequently, the optimum adsorbent dose was set at 50 mg for the remainder of the work.

The removal of Cu^2+^ and Ni^2+^ cations in contact with alginate-Moroccan clay bio-nanocomposite beads indicates that the Cu^2+^ and Ni^2+^ cation solutions show a higher adsorption capacity up to the value of ratio R = 1 g/L (50 mg/50 mL), and that any further addition of alginate-Moroccan clay bio-nanocomposite beads does not show a significant increasing effect on the retention process. This result can be interpreted as meaning that a high mass of adsorbent creates agglomerations of particles, which lowers the amount of adsorbate per unit mass of adsorbent and decreases the overall adsorption surface area [38,47,48].

#### 3.2.2. Effect of Contact Time

**Figure 6:**
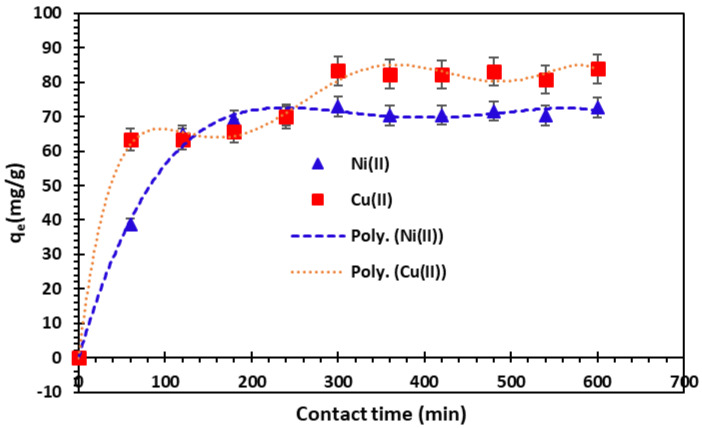
Effect of contact time on the removal of Cu^2+^ and Ni^2+^ cations onto bio-nanocomposite beads (*m*/*V* = 1 g/L and *T* = 25 °C; error bars show means ± standard error from the mean of duplicate experiments).

To establish an appropriate contact time between the adsorbent and solution metal ions, the adsorption capacities in time were measured. The results depicted in Figure 6 show that the removal of Cu^2+^ and Ni^2+^ ions is most likely occurring in two steps.

The first step is relatively rapid, and the second indicates the achievement of the equilibrium. This time is largely sufficient to establish equilibrium to study the parameters affecting the removal of Cu^2+^ and Ni^2+^ cations by the studied adsorbent. When equilibrium is established, the adsorption rate is stable. The rapid step is probably due to the high availability of the active sites on the alginate-Moroccan clay bio-nanocomposite beads’ surface, the adsorption becomes progressively less efficient in the second slower step due to the progressive occupancy of highly active sites [47,49]. For alginate-Moroccan clay bio-nanocomposite beads, the amount of Cu^2+^ and Ni^2+^ adsorbed stabilizes at a contact time of 300 min. After adsorption reached equilibrium, the adsorption capacities of Ni^2+^ and Cu^2+^ were of 72.72 mg/g and 83.30 mg/g at 300 min. This high level of adsorption could be explained by the existence of readily available reactive sites on the outer surface of the bio-nanocomposite beads, which facilitated the removal of Cu^2+^ and Ni^2+^ cations during the initial phase [50].

A similar study was carried out by Benhima et al. (2011) [47] on the elimination of metallic ions such as Pb^2+^, Zn^2+^, Cd^2+^, and Cu^2+^ cations by microparticles of the W. frutesens plant as an adsorbent. Benhima et al. (2011) observed that the adsorption of the metals studied takes place in two stages. The first stage involves rapid metal uptake, the second stage is characterized by adsorption equilibrium at 300 minutes.

Barrak et al. (2022) [38] carried out a similar kinetics study of Cu^2+^ adsorption on alginate-encapsulated clay beads and showed that, after 190 min, the adsorption capacity of Cu^2+^ was 60.05 mg/g. 

#### 3.2.3. Effect of pH Solution

**Figure 7:**
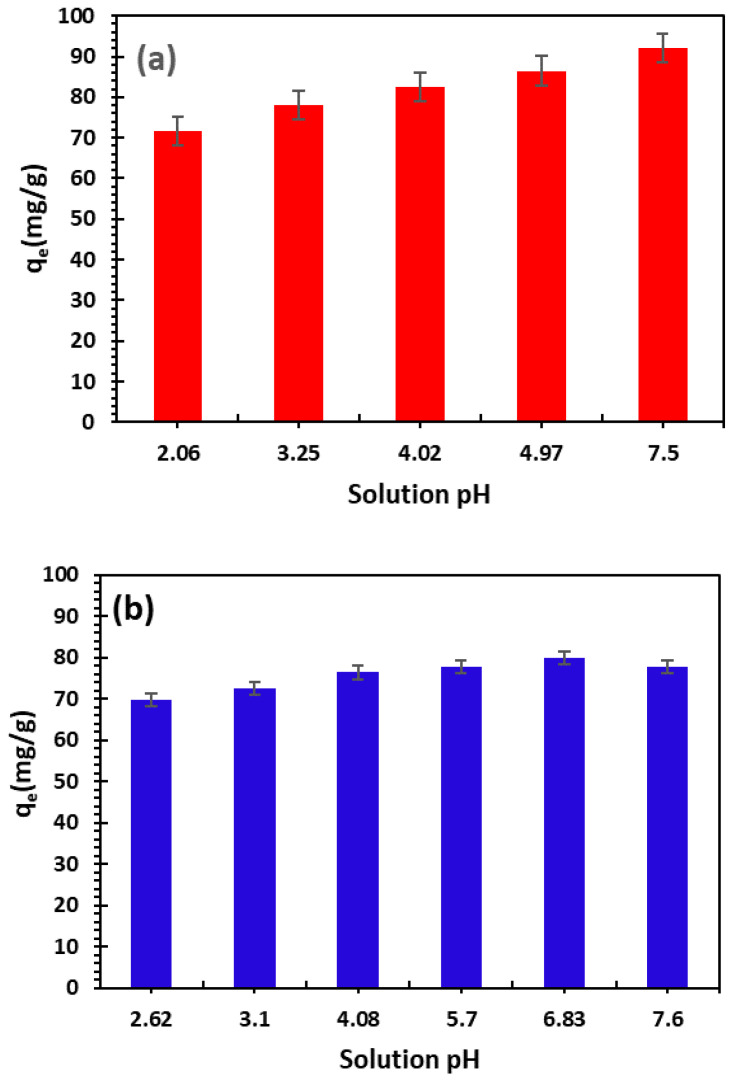
Effect of initial pH on the adsorption of Cu^2+^ (**a**) and Ni^2+^ (**b**) cations (*C*_0_ = 100 mg/L, *m*/*V* = 1 g/L and *T* = 23 ± 2 °C; error bars show means ± standard error from the mean of duplicate experiments).

The following methods were used to study the effect of pH. In the first step, 0.05 g of bio-nanocomposite beads were filled into several flasks and the process was studied at different pH values with an initial concentration of 100 mg/L. The pH ranged from 2.06 to 7.5 for Cu^2+^ and from 2.62 to 7.5 for Ni^2+^. As indicated in Figure 7, increasing the pH leads to an increase in the adsorption capacity.

The low augmentation may indicate that the Moroccan alginate-clay bio-nanocomposite used in this study is insensitive to pH solution variations, because the alginate gel structure helps to maintain a stable environment around the clay particles, preventing pH variations from affecting the adsorption capacity of the material.

We can see that, at pH > pH_ZPC_, the increase in adsorption capacity is attributed to electrostatic attraction between the cations and the negatively charged adsorbent surface [21,51]. 

A similar behavior was observed by Stefan and Meghea (2014) [52] for the removal of Ca^2+^, Pb^2+^, and Ni^2+^ cations using Purolite1 S930 ion exchange resin. The authors supposed that, with the pH increase, the proton concentration inside the aqueous medium was lower. 

#### 3.2.4. Effect of Temperature

**Figure 8:**
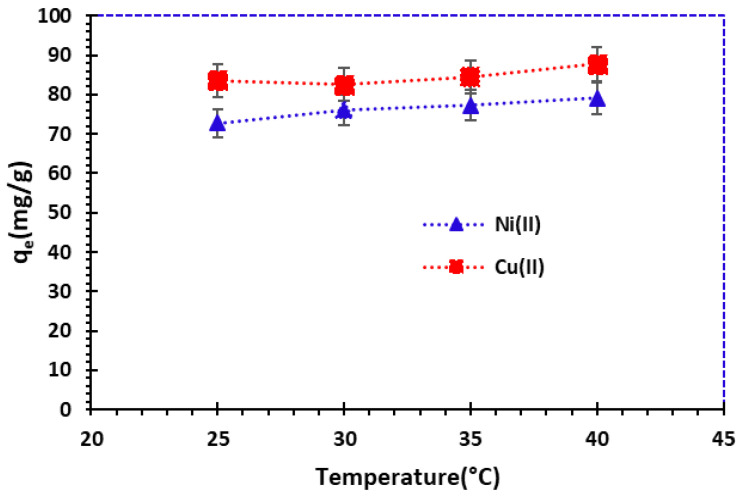
Effect of temperature on adsorption of Cu^2+^ and Ni^2+^ cations by bio-nanocomposite beads (*C*_0_ = 100 mg/L and *m*/*V* = 1 g/L; error bars show means ± standard error from the mean of duplicate experiments).

Figure 8 shows the temperature influence on the metal ions’ removal, with an initial concentration of 100 mg/L. According to Figure 8, a slight increase in adsorption capacity was observed when the temperature was increased from 25 °C to 40 °C. The adsorbed amount was set at 83.64 mg/g for Cu^2+^ and 72.73 mg/g for Ni^2+^ at 25 °C.

It can be concluded that the quantity of adsorption is increasing with temperature, implying endothermic adsorption. These findings can be verified by determining the thermodynamic parameters.

An equivalent study was carried out by Alothman et al. (2020) [53] on the adsorption of metal ions (Cu^2+^, Pb^2+^, and Cd^2+^ cations) by low-cost bio-sorbents from fungi. This study showed that the adsorption capacity increases from 10 °C to 60 °C. The bio-sorption efficiency increased, due to greater affinity of the active sites, leading to greater attraction of heavy metal ions. 

### 3.3. Adsorption Kinetic Models

The retention process consists of the following three steps:

- External diffusion, which means the mass transfer of the adsorbate from the bulk solution to the external surface of the adsorbent;
- Internal diffusion of the adsorbate through the pores of the adsorbent;
- The adsorption itself on the active centers of the adsorbent.

The slowest step among the three steps is the rate-limiting step. This can be elucidated by fitting the experimental data with different kinetic models to establish the most probable adsorption mechanism. Four kinetic models have been studied in this context [21].

#### 3.3.1. Pseudo-First-Order Kinetics Model

The kinetic equations below show the linear and non-linear form of the pseudo-first-order model [21,52,53,54,55,56]:

(3) $$\frac{dq_{t}}{dt}=k_{1}\left(q_{e}-q_{t}\right)\;\;\;\;\left(non-linear\;form\right)\;\;\;\;\;$$

(4) $$\ln\left(q_{e}-q_{t}\right)=\ln q_{e}-k_{1}t\;\;\;\;\;\left(linear\;form\right)$$

In the pseudo-first-order kinetics model, where *k*_1_ (min^−1^) stands for the rate constant, *q_e_* (mg/g) and *q_t_* (mg/g) denote the removed amounts of Cu^2+^ and Ni^2+^ cations per unit mass of bio-nanocomposite can reach equilibrium at any time t(min) respectively.

**Figure 9:**
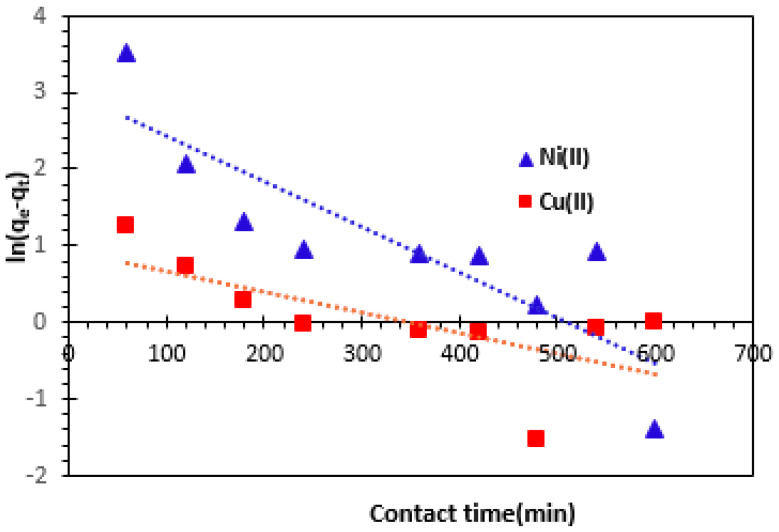
Pseudo-first-order adsorption kinetics model of Cu^2+^ and Ni^2+^ retention by bio-nanocomposite beads.

The graph of *ln(q_e_−q_t_)* against contact time (t) for bio-nanocomposite beads results in a linear plot with a slope of -*k*_1_ and intercepts *ln qe* (Figure 9). Table 2 provides the values for the theoretical adsorption capacity (*q_e_, _Theo_*), the rate constant for the pseudo-first-order kinetics model (*k*_1_), and the correlation coefficient (*R*^2^). The table shows that the value of the theoretical adsorbed amount *q_e_* is different to the experimental value (*q_e, Theo_* = 20.46 mg/g < *q_e, Exp_* = 72.82 mg/g for Ni^2+^ and *q_e, Theo_* = 2.55 mg/g < *q_e, Exp_* = 83.30 mg/g for Cu^2+^, suggesting that the pseudo-first-order model does not fit well the experimental data. 

We can see that, under such conditions, the pseudo-first-order model is not suitable for describing the adsorption kinetics of Cu^2+^ and Ni^2+^ cations onto the prepared bio-nan-composite. 

#### 3.3.2. Pseudo-Second-Order Kinetics Model

The linear and non-linear forms of the pseudo-second-order kinetic model are illustrated as below [21,54,55,56,57,58,59]:

(5) $$\frac{dq_{t}}{dt}=k_{2}{\left(q_{e}-q_{t}\right)}^{2}\;\;\left(non-linear\;form\right)\;\;\;\;$$

(6) $$\frac{t}{q_{t}}=\frac{1}{k_{2}q_{e}^{2}}+\left(\frac{1}{q_{e}}\right)t\;\;\;\;\;\left(\;linear\;form\right)$$

where *k*_2_ (g.mg^−1^**.**min^−1^) is the rate constant of the pseudo-second-order kinetic model.

**Figure 10:**
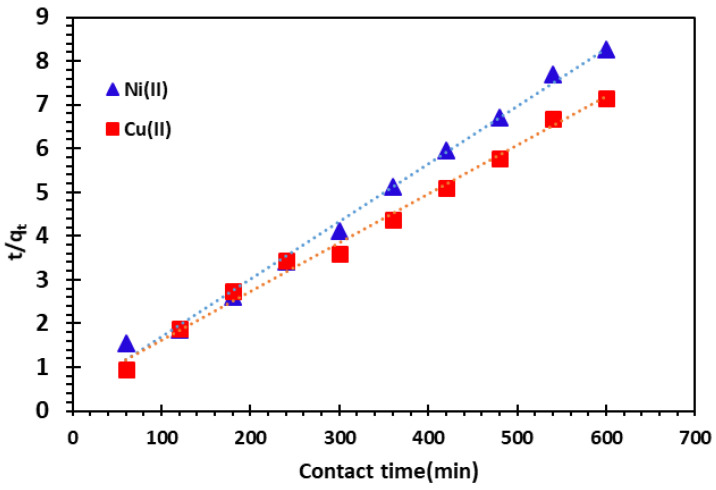
Pseudo-second-order kinetics model of Cu^2+^ and Ni^2^ cations adsorption on bio-nanocomposite beads.

The pseudo-second-order kinetics model results for the adsorption of Cu^2+^ and Ni^2+^ cations are shown in Figure 10, and the kinetic parameters are listed in Table 2. The results shown in Table 2 indicate that the correlation coefficient *R*^2^ of the pseudo-second-order kinetics model is approaching equal to 1, and the theoretical adsorption capacity *(q_e, the_*) is similar to the experimental value (*q_e, Theo_* = 75.75 mg/g for Ni^2+^ and *q_e, Theo_* = 90.09 mg/g for Cu^2+^).

Therefore, the pseudo-second-order adsorption model is more suitable to describe the adsorption kinetics of Cu^2+^ and Ni^2+^ cations on the alginate-Moroccan clay bio-nanocomposite beads.

#### 3.3.3. Elovich Kinetic Model

The Elovich model is a useful tool for studying systems with heterogeneous surfaces, especially when describing the kinetics of chemisorption [21]. This model is mathematically expressed through Equations (7) and (8). In these equations, *“q_e_”* and “*q_t_*” (measured in mg/g) denote the quantities of adsorbed Cu^2+^ and Ni^2+^ cations at equilibrium, and at any specific contact time “t” (in minutes), respectively.

The kinetics equation Elovich model and its linearized form may be expressed as the following:

(7) $$\frac{dq_{t}}{dt}=\alpha e^{-\beta q_{t}}\;\;\;\left(non-linear\;form\right)\;\;$$

**Figure 11:**
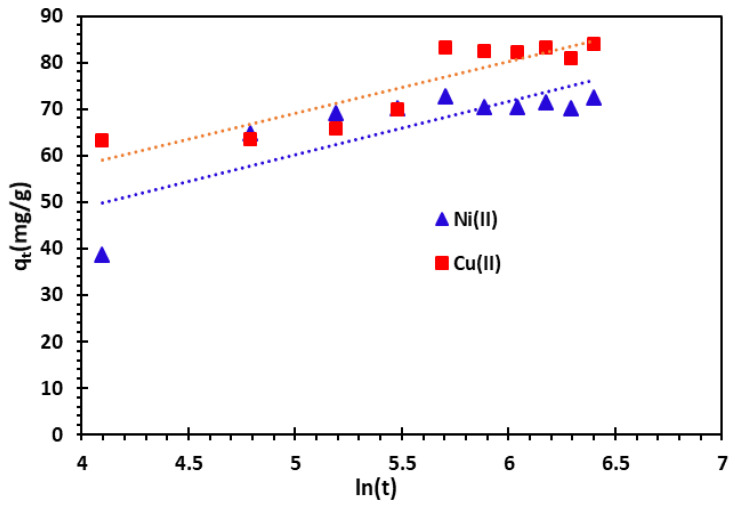
Elovich kinetic model of Cu^2+^ and Ni^2+^ cations adsorption on bio-nanocomposite beads.

(8) qt=ln⁡(αβ)β+1β ln⁡(t)   linear form

where *β* (g/mg) is the desorption constant associated with the extent of the surface coverage and chemisorption activation energy. α (mg/g/min) is the initial adsorption rate. The kinetic Elovitch constants *α* and *β* are determined from the intercept and slope, respectively (Figure 11). The *R*^2^ values (correlation coefficient) designate that this model is not suitable for characterizing the removal of metal cations on bio-nanocomposite beads. However, based on the correlation coefficient analysis, it is obvious that the Elovich model is not sufficiently accurate to characterize the removal of metal cations on bio-nanocomposite.

#### 3.3.4. Intra-Particle Diffusion Kinetics Model

Fitting the experimental data to an intraparticle diffusion model is the most widely used method for determining the mechanism involved in the sorption process. One or more processes, such as boundary layer (film) or external diffusion, diffusion at the surface, internal pore diffusion, or a combination of several steps, may be used to approximate the overall adsorption of solute onto the solid surface [21,60]. 

**Figure 12:**
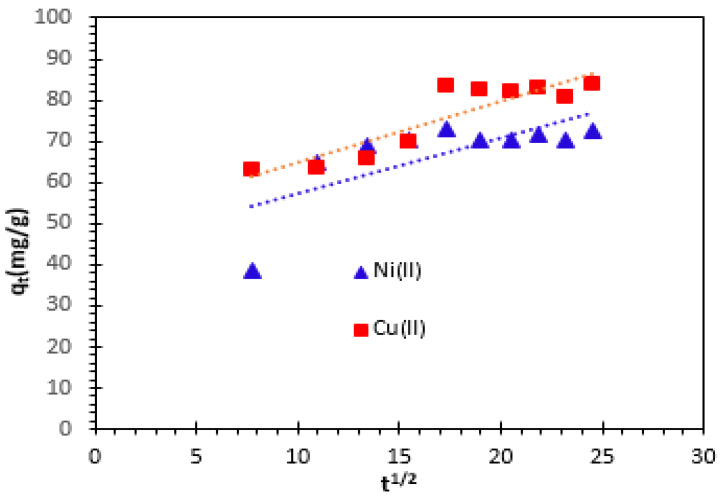
Intra-particle diffusion kinetics model of Cu^2+^ and Ni^2^ cations adsorption on bio-nanocomposite beads.

Equation (9) becomes linear to determine the initial intra-particle diffusion rate. This constant is given by *k_p_* (mg^−1^_·_min^1/2^), and the amount of Cu^2+^ and Ni^2+^ cations removed per unit weight of adsorbent at contact time t (min) is given by *q_t_* (mg/g). The results are presented in Figure 12.

(9) $$q_{t}=k_{p}t^{1/2}+c\;\;\;\;\left(linear\;form\right)\;\;$$

The *kp* value for intraparticle diffusion is obtained from the slope of the straight line portion of the *q_t_* versus *t*^1/2^ plot at different solution temperatures. At 25 °C, the correlation coefficients (*R*^2^) of the two cations detected were 0.815 for Ni^2+^ and 0.530 for Cu^2+^. This correlation coefficient denotes that the intraparticle diffusion model is not satisfactory to describe the kinetics of adsorption of Cu^2+^ and Ni^2+^ cations from aqueous solution onto bio-nanocomposite beads. This means that the internal diffusion is either fast and is not the limiting step in the adsorption mechanism or internal diffusion is not solely the limiting step. The values of *k_p_* and *c* calculated from the slopes and intercepts are summarized in Table 2.

The investigation into the retention of Cu^2+^ and Ni^2+^ cations using the bio-nanocomposite showed exceptionally high correlation coefficient (*R*^2^) values, approximately 0.99, for the pseudo-second-order adsorption kinetics model, particularly when the initial concentration was 100 mg/L.

This means that the pseudo-second-order model’s estimation of adsorption capacity nearly matched the experimental results. The pseudo-second-order adsorption model is thought to be the best option for explaining the kinetics of Cu^2+^ and Ni^2+^ cations’ adsorption by the used bio-nanocomposite.

### 3.4. Isotherm Study

Adsorption isotherms serve as crucial tools for comprehending the adsorption mechanism, providing models that elucidate the distribution of adsorbed species between the solid and liquid phases [61]. In this study, various mathematical models were examined, including the Langmuir, Freundlich, Temkin, and Dubinin-Radushkevich (D-R) equations. This study was performed by ranging the initial concentration of copper and nickel ions from 100 to 600 mg/L, at room temperature.

#### 3.4.1. Langmuir Adsorption Isotherm

According to the Langmuir adsorption isotherm, a homogeneous surface is represented by a solid surface with a limited number of identic sites [21,61,62,63,64]. The nonlinear and linearized forms of the Langmuir equation are given by the Equations (10) and (11):

(10) $$q_{e}=q_{L}\frac{K_{L}C_{e}}{1+K_{L}C_{e}}\;\;\;(non-linear\;form)$$

(11) $$\frac{1}{q_{e}}=\frac{1}{q_{L}}+\frac{1}{q_{L}K_{L}C_{e}}\;\;\;\;\;\;(linear\;form)\;\;\;\;$$

where *q_e_* (mg/g) is the amount adsorbed at equilibrium concentration *C_e_* (mg/L), *q_L_*(mg/g) is the Langmuir maximum removed amount, and *K_L_* (L/mg) is the Langmuir constant related to energy of adsorption. 

**Figure 13:**
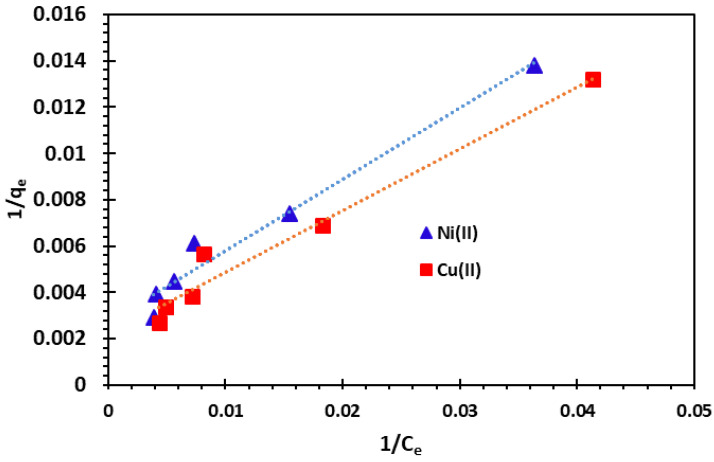
Langmuir adsorption isotherm of Cu^2+^ and Ni^2+^ cations at 25 °C.

The plots between 1*/q_e_* and 1*/C_e_* for the adsorption of Cu^2+^ and Ni^2+^ cations are represented in Figure 13. 

The basic characteristics of the Langmuir isotherm can be supposed by “R_L_”, which is a dimensionless constant called a separation factor or equilibrium parameter that is used to predict whether an adsorption system is favourable or not. *R_L_* is calculated using Equation (12), where *K_L_* (L/mol) is the Langmuir constant and *C*_0_ (mol/L) is the highest initial ion concentration.

(12) $$R_{L}=\frac{1}{1+K_{L}C_{0}}$$

The calculated values of parameter *R_L_* for this study were found to be between 0 and 1 (0.160 for Ni^2+^ and 0.168 for Cu^2+^ ions), indicating that the adsorption of Cu^2+^ and Ni^2+^ cations onto bio-nanocomposite bead particles was favourable (Table 3).

The values of the adsorption capacity (*q_L_*), the Langmuir constant (*K_L_*), and the correlation coefficient (*R*^2^) were presented in Table 4. The highest value of the adsorption capacity *q_L_* obtained at 25 °C was 370.37 mg/g for Ni^2+^ and 454.54 mg/g for Cu^2+^ ions (Table 4).

#### 3.4.2. Freundlich Adsorption Isotherm

For natural adsorbents which are heterogeneous materials, the Freundlich equation offers the most suitable adsorption data. The Freundlich adsorption isotherm equations in non-linear and in its linear forms are [21,61,62,63]:

(13) $$q_{e}=K_{F}{C_{e}}^{1/n}\;\;\;\;\;\;\;\;\;\;\;\left(non-linear\;form\right)\;\;\;\;\;\;\;\;\;$$

(14) $$\ln q_{e}=\ln K_{F}+\frac{1}{n}\ln C_{e}\;\;\;\;\;\;\;\left(linear\;form\right)$$

In the given context, *q_e_* (mg/g) means the amount of metal cations eliminated per unit mass of adsorbent. *C_e_* (mg/L) represents the equilibrium concentration of solute in the total solution. *K_F_* (mg/g) is the Freundlich constant, which is a relative indicator of the adsorption capacity of an adsorbent. The parameter “*n*” is an empirical constant related to the heterogeneity of the adsorbent surface and also indicates the nature of the adsorption process.

**Figure 14:**
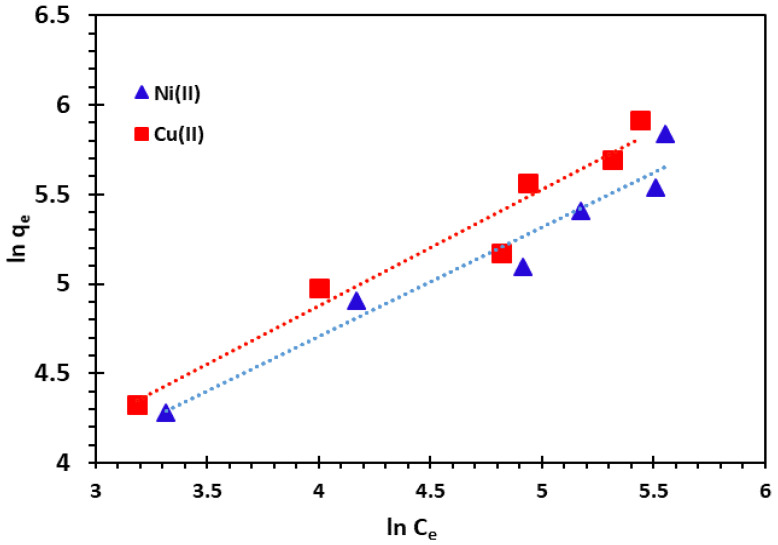
Freundlich adsorption isotherm of Cu^2+^ and Ni^2+^ cations at 25 °C.

When the value of 1/*n* is between 0 and 1, adsorption is favourable, whereas if its value is 0, this means that the surface can be more heterogeneous in nature [65,66]. The slope and intercept of the plot ln *q_e_* vs ln *C_e_* were used to obtain the isotherm constants *n* and *K_F_* (Figure 14). The values of Freundlich constants and *R*^2^ are also presented in Table 4 for both temperatures.

The Freundlich isotherm constants K_F_ and *n* are constants that take into account all factors affecting the adsorption process, such as adsorption capacity and adsorption intensity. The constants *K_F_* and *n* were calculated from Equation (14).

These experiments confirm the effectiveness of the bio-nanocomposite beads used to remove Cu^2+^ and Ni^2+^ cations from aqueous solutions.

#### 3.4.3. Temkin Isotherm

**Figure 15:**
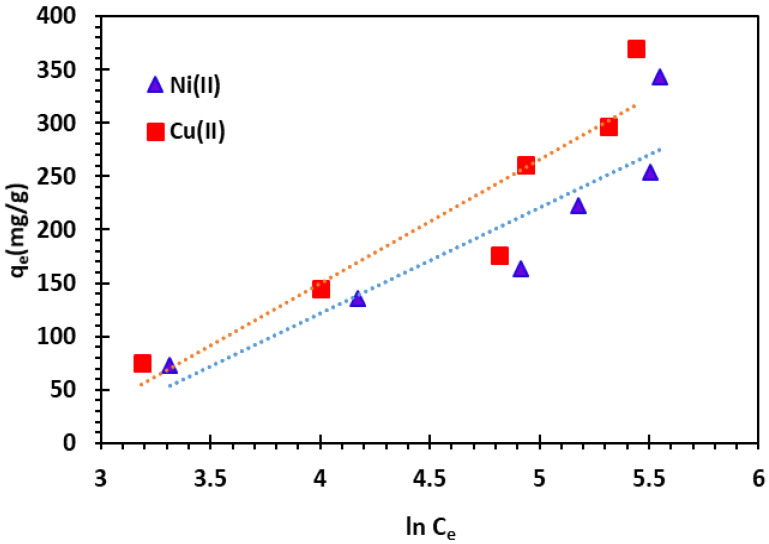
Temkin adsorption isotherm of Cu^2+^ and Ni^2+^ cations at 25 °C.

Temkin’s adsorption isotherm model is designed around the heat of ion adsorption, generated by the interaction between adsorbate and adsorbent. The Temkin isotherm equation is written as follows [21,67]:

(15) $$\ln q_{e}=\ln K_{F}+\frac{1}{n}\ln C_{e}\;\;\;\;\;\;\;\left(linear\;form\right)$$

(16) $$q_{e}=\frac{RT}{b_{T}}\ln K_{T}+\frac{RT}{b_{T}}\ln C_{e}\;\left(\;linear\;form\right)\;\;\;$$

where *T* is the temperature in Kelvin and *R* the universal gas constant (8.314 J/mol/K). *b_T_* (J/mol) is the Temkin isotherm constant related to the adsorption heat. *K_T_* (L/mg) is the equilibrium binding constant corresponding to the maximum binding energy. The Temkin isotherm plot is illustrated in Figure 15. The isotherm parameters are given in Table 4. 

The Temkin constants *b_T_* linked to heat of adsorption of Cu^2+^ and Ni^2+^ cations at 25 °C were found to be 24.938 J/mol for Ni^2+^ and 21.33 J/mol for Cu^2+^, respectively.

The linear regression of the experimental data shows rather low *R*^2^ values (0.829 for Ni^2+^ and 0.865 for Cu^2+^), indicating that the adsorption of Cu^2+^ and Ni^2+^ cations does not fully follow the Temkin isotherm.

#### 3.4.4. Dubinin-Radushkevich (D-R) Isotherm

When expressing the adsorption mechanism with a Gaussian energy distribution onto a heterogeneous surface, the Dubinin-Radushkevich isotherm is usually used. It is used for calculating the mean free energy of adsorption *(E*), not the constant adsorption potential or homogenous surface assumption. The D-R equation can be written in both linear and non-linear versions as follows [21]:

(17) $$q_{e}=q_{m}e^{{-K}_{p}\varepsilon^{2}}\;\;\left(non-linear\;form\right)$$

(18) $$\ln q_{e}=\ln q_{m}{-K}_{D}\varepsilon^{2}\;\;\left(linear\;form\right)$$

where *q_m_* (mg/g) is the theoretical saturation capacity and *ε* is the Polanyi potential that can be calculated from Equation (19):

(19) $$\varepsilon =RT\ln\left(1+\frac{1}{C_{e}}\right)\;\;\;\;$$

When the adsorbate is moved from the bulk solution to the surface of the solid, the constant *K_D_* (mol^2^/J^2^) provides an estimate of the mean free energy *E* (kJ/mol) of adsorption per molecule. This may be computed from the *K_D_* value using the following relation (Equation (20)):

(20) $$E=\frac{1}{{\left(2K_{D}\right)}^{1/2}}$$

**Figure 16:**
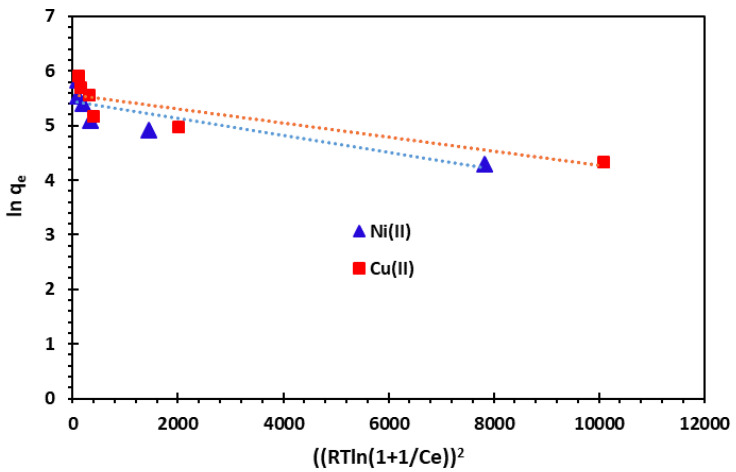
Dubinin–Radushkevich (D–R) adsorption isotherm of Cu^2+^ and Ni^2+^ cations at 25 °C.

When the value of E falls within the range of 8 to 16 kJ/mol, it suggests that the adsorption process is likely chemisorption. Conversely, for values of E less than 8 kJ/mol, the adsorption process is expected to be of a physical nature (Figure 16). These outcomes are depicted in Table 4. 

The slope of the *ln q_e_* versus ε^2^ plot provides the dissociation constant *K_D_*, while the intercept yields the adsorption capacity *q_m_*. In Table 4, the correlation coefficient values are observed to be 0.756 for Ni^2+^ and 0.771 for Cu^2+^ at 25 °C. The numerical value of the mean free energy of adsorption is 50 J/mol for Ni^2+^ and 70.71 J/mol for Cu^2+^. These values are indicative of physisorption, suggesting the predominance of van der Waals forces in the adsorption process.

### 3.5. Thermodynamic Study

Determining the thermodynamic parameters is essential for comprehending the link between temperature and adsorption, which is mostly dependent on the specific combination of adsorbent and adsorbate. Typically, adsorption is accompanied by a thermal effect, manifesting as either exothermic (Δ*H*° < 0) or endothermic (Δ*H*° > 0). The assessment of the heat change (Δ*H*°) plays a crucial role as the principal indicator for discerning between chemisorption and physisorption. Moreover, the calculation of the standard entropy change (Δ*S*°) helps in gauging the level of disorder within the adsorbate-adsorbent system. Additionally, the assessment of the standard Gibbs free energy change (Δ*G*°) enables us to predict the spontaneity of the process [21,51,67,68]. These thermodynamic parameters were calculated from the following equations:

(21) $$∆G{}^{\circ}=-RTLn\left(K_{d}\right)$$

(22) $$ln\left(K_{d}\right)=-\frac{∆H{}^{\circ}}{RT}+\frac{∆S{}^{\circ}}{R}$$

(23) ∆G° =∆H°−T∆S°

where *T* is the absolute temperature in Kelvin, *R* is the universal gas constant (8.314 J/mol/K), and *K_d_* (L/mol) represents the distribution coefficient. Table 5 shows the results of the thermodynamic parameters. From the overall results, we can conclude that the positive values of Δ*H*° indicate that the adsorption process is physical and endothermic, which is consistent with the experimental data. Negative ∆*G*° values demonstrate the spontaneous nature of the adsorption process. Positive Δ*S*° values reveal increasing randomness at the solid/liquid interface during metal ion adsorption onto the prepared bio-nanocomposite.

### 3.6. Binary Component Systems Adsorption

**Figure 17:**
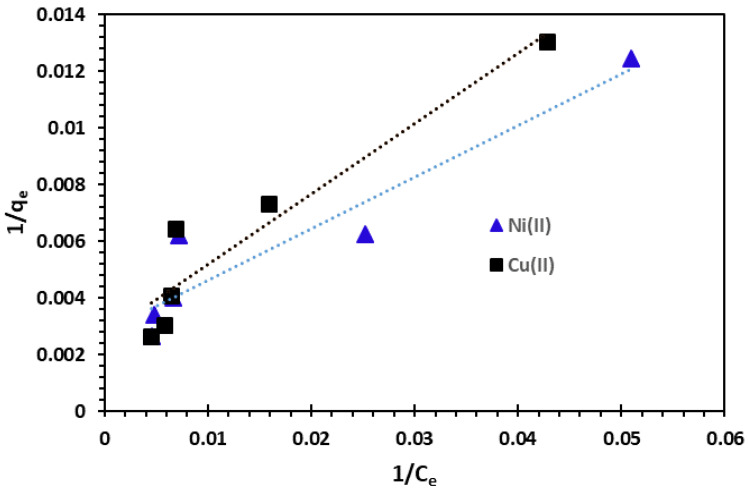
Langmuir isotherm of Ni^2+^ and Cu^2+^ cations on bio-nanocomposite beads in binary component system.

**Figure 18:**
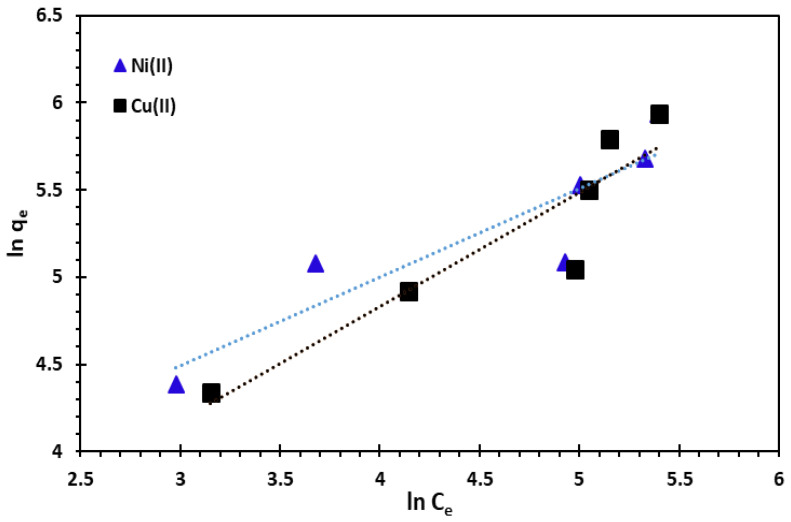
Freundlich isotherm of Ni^2+^ and Cu^2+^ cations on bio-nanocomposite beads in binary component system.

To determine the mechanism of Cu^2+^ and Ni^2+^ cations’ removal from binary component system on bio-nanocomposite beads, the experimental data were fitted using Langmuir and Freundlich isotherm equations. The experimental data were obtained at room temperature, at the initial ion pH solutions, and with percentages of 50% Ni^2+^ + 50% Cu^2+^. The results are shown in Figure 17 and Figure 18.

Table 6 displays the values of the Freundlich and Langmuir parameters for each cation in the binary system along with the correlation coefficient (*R*^2^) values.

The interactions between both metal ions in the binary mixture were evaluated by the ratio *q_L, mix_/ q_L, single_, q_L, mix_*, which is the maximum adsorption capacity in the binary mixture, and *q_L, single_* is the maximum adsorbed amount in the single system [69,70].

- *q_L, mix_/ q_L, single_* > 1: adsorption is promoted by the presence of other ions;
- *q_L, mix_/ q_L, single_* ˂ 1: adsorption is suppressed by other ions;
- *q_L, mix_/ q_L, single_* = 1: there is no visible net interaction.

This work indicates that, if we mixed two metal ions, the ratio *q_L, mix_/ q_L, single_* is less than 1, which shows a great competition between both the ions (Ni^2+^ + Cu^2+^) to occupy the active sites (antagonism effect).

### 3.7. Adsorption Mechanisms

**Figure 19:**
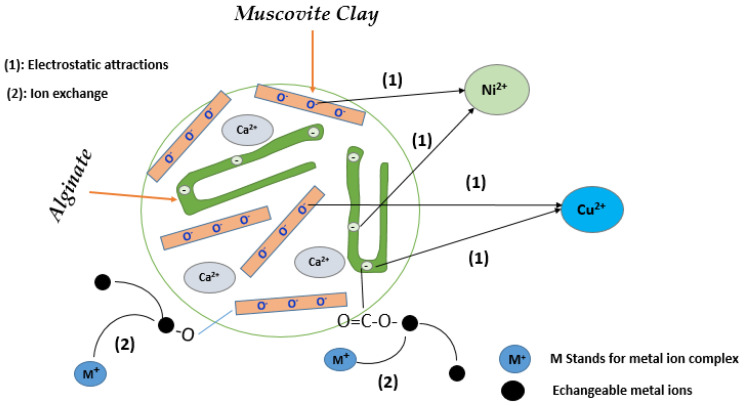
Adsorption mechanisms of alginate-Moroccan clay adsorbent for metal ions.

The adsorption mechanism is very important in order to obtain an idea of what may be responsible for the removal of metal ions by the bio-adsorbent used in this study (Figure 19). 

To improve production processes and optimize the practical applications of bio-composites, a deeper understanding of the mechanisms governing the adsorption of Cu^2+^ and Ni^2+^ ions is crucial. This category encompasses the majority of investigated bio-composites. As depicted in Figure 19, the fundamental mechanisms include electrostatic attractions and ion exchange. These mechanisms play a crucial role in comprehending the adsorption of metal ions onto bio-nanocomposites. Electrostatic attraction serves as a vital initial step, facilitating the interaction between the anionic functions of positively charged Cu^2+^ and Ni^2+^ ions and the negatively charged sites on the surface of the bio-nanocomposite [71].

### 3.8. Comparison with Previous Literature Data

To justify the validity of alginate-Moroccan clay bio-nanocomposite for adsorption processes, its adsorption potential must be compared with other various adsorbents used for this purpose. The theoretical amount adsorbed values of the pseudo-second-order kinetic model for the removal of Cu^2+^ and Ni^2+^ metal ions reported in the literature are given in Table 7. The direct comparison of the theoretical amount adsorbed of metal ions by alginate-Moroccan clay bio-nanocomposite with other adsorbents capacity reported in the literature is difficult due to the different experimental conditions employed in those studies (initial concentration, ratio m/V, pH of solution, and contact time). 

However, alginate-Moroccan clay bio-nanocomposites in this study possess reasonable adsorption capacity in comparison with other adsorbents.

## 4. Conclusions

The present work aims to characterize and evaluate the removal of Cu^2+^ and Ni^2+^ from single and binary systems by alginate-Moroccan clay bio-composite with the use of calcium chloride as a cross-linking agent, using the ionotropic gelation method. The results of the study demonstrated that the adsorption process is represented by second-order kinetics, and the associated kinetic parameters were found. 

The adsorption equilibrium was explored through various mathematical models, including the Langmuir, Freundlich, Temkin, and Dubinin-Radushkevich isotherm models, to assess the characteristics associated with the adsorption process. The Langmuir isotherm emerged as the most fitting model for representing the adsorption of Cu^2+^ and Ni^2+^ ions using bio-nanocomposite beads. The bio-nanocomposite beads demonstrated a maximum adsorption capacity of 370.37 mg/g for Ni^2+^ and 454.54 mg/g for Cu^2+^ in the single system. In the binary system, the maximum adsorption capacities were observed to be 357.14 mg/g for Ni^2+^ and 370.37 mg/g for Cu^2+^. 

The process is considered to be physical and endothermic, as suggested by the positive values of Δ*H*° and Δ*S*°, indicating in-creased randomness at the solid/liquid interface during Cu^2+^ and Ni^2+^ adsorption. Furthermore, the spontaneity of the process is confirmed by the negative values of ∆G°. This provides compelling evidence that alginate-Moroccan clay bio-nanocomposites can serve as cost-effective adsorbents, effectively removing metal ions such as Ni^2+^ and Cu^2+^ from both single and binary systems.

## Figures and Tables

**Table 1:** EDX analysis results of bio-nanocomposite beads.

Elements	Atomic Percentage (%)
**C**	33.83
**O**	54.04
**Na**	0.76
**Mg**	0.46
**Al**	3.55
**Si**	5.45
**Ca**	1.00
**S**	0.14
**K**	0.29
**Ti**	0.23

**Table 2:** Parameters of four kinetic models for Cu^2+^ and Ni^2+^ cations’ removal at *C*_0_ = 100 mg/L.

Kinetics Model	Parameters	Metal Ions	
		Ni^2+^	Cu^2+^
*Pseudo- first-order model*	*q_e, Exp_* (mg/g)	72.82	83.30
	*K*_1_ (min^−1^)	0.0059	0.0027
	*q_e, Theo_* (mg/g)	20.46	2.55
	*R* ^2^	0.737	0.469
*Pseudo-second-order model*	*q_e, Exp_* (mg/g)	72.82	83.30
	*K*_2_ (g.mg^−1^. min^−1^)	4.5 × 10^−4^	2.5 × 10^−4^
	*q_e, Theo_* (mg/g)	75.75	90.09
	*R* ^2^	0.994	0.992
*Elovich model*	*q_e, Exp_* (mg/g)	72.82	83.30
	*α*	14.64	38.94
	*β*	0.087	0.090
	*R* ^2^	0.679	0.806
*Intra-particle diffusion*	*K_Int_* (mg.g^−1^. min^−1/2^)	1.48	1.35
	*C_I_*	50.028	43.52
	*R* ^2^	0.815	0.530

**Table 3:** Isotherm type for various R_L_ values.

Dimensionless Constant	Metal Ions	
	Ni^2+^	Cu^2+^
*R_L_*	0.160	0.168

**Table 4:** Parameters of isotherm models for Cu^2+^ and Ni^2+^ cations’ adsorption at 25 °C.

Model	Parameters	Metal Ions	
		Ni^2+^	Cu^2+^
Langmuir	*q_L_* (mg/g)	370.37	454.54
	*K_L_* (L/mg)	0.0087	0.0082
	*R* ^2^	0.970	0.970
Freundlich	*1/n*	0.608	0.650
	*K_F_* (mg/g)	9.706	9.728
	*R* ^2^	0.946	0.950
Temkin	*K_T_* (L/mg)	0.062	0.066
	*b_T_* (J/mol)	24.94	21.33
	R²	0.829	0.865
D-R	*K_D_* (mol^2^/J)	2 × 10^−4^	10^−4^
	*q_m_* (mg/g)	229.63	258.60
	*E* (J/mol)	50	70.71
	R²	0.756	0.771

**Table 5:** Thermodynamic parameters for of Cu^2+^ and Ni^2+^ cations at 25 °C adsorption at *C*_0_ = 100 mg/L.

Ion	∆*G°* (kJ/mol)				∆*H°* (kJ/mol)	∆*S°* (J/K/mol)
	298 K	303 K	308 K	313 K		
Ni^2+^	−12.52	−13.18	−13.56	−14.06	17.178	99.83
Cu^2+^	−14.33	−14.71	−15.69	−16.71	33.89	161.15

**Table 6:** Parameters of Langmuir and Freundlich models for the adsorption of Ni^2+^ and Cu^2+^ cations on bio-nanocomposite beads in binary component system.

System	Freundlich Parameters			Langmuir Parameters			$\frac{q_{L,mix}}{q_{L,Single}}$
	1/*n*	$K_{f}\;(mg/g)$	$R^{2}$	$q_{L}$ (mg/g)	$K_{L}\;(L/mg)$	$R^{2}$	
Ni^2+^ Cu^2+^	0.507	19.53	0.823	357.14	0.0015	0.892	0.96
	0.655	9.09	0.853	370.37	0.010	0.902	0.81

**Table 7:** Theoretical amount adsorbed of metal ions by other natural adsorbents compared with alginate-Moroccan clay Bio-nanocomposite.

Adsorbent	Metal Ion	*C*_0_ (mg/L)	*q_e, Theo_* (mg/g)	Reference
Alginate immobilized bentonite	Cu^2+^	100 100 100	60.24	[72]
Sodium alginate encapsulated Moroccan clay	Cu^2+^		62.620	[38]
Moroccan clay			35.530	
Chitosan immobilized on bentonite	Pb^2+^	200	28.65	[73]
	Cu^2+^		20.28	
	Ni^2+^		11.83	
Alginate-Moroccan clay bio-nanocomposite	Cu^2+^	100	90.09	This study
	Ni^2+^		75.75	

## Data Availability

Data can be provided upon request.
